# Case report: One child with an autism spectrum disorder who had chronically elevated serum levels of CK and CK-MB

**DOI:** 10.3389/fpsyt.2022.995237

**Published:** 2022-09-06

**Authors:** Ping Rong, Shuyi Zhao, Qianfang Fu, Mengrui Chen, Libin Yang, Yifei Song, Xilian Zhang, Rong Ma

**Affiliations:** ^1^First Teaching Hospital of Tianjin University of Traditional Chinese Medicine, Tianjin, China; ^2^National Clinical Research Center for Chinese Medicine Acupuncture and Moxibustion, Tianjin, China

**Keywords:** autism (ASD), creatine kinase, biomarkers, traditional Chinese medicine, case report

## Abstract

Some patients with autism spectrum disorder (ASD) exhibit elevated serum creatine kinase levels, which are believed to be associated with mitochondrial dysfunction. Although a few articles have reported this situation in the past and the increase mostly ranges from 100 to 300 U/L, there is a paucity of previous study focusing on the serum creatine kinase MB isoenzyme. This article discusses a 5-year-old girl with ASD, whose serum creatine kinase and creatine kinase MB isoenzyme have been rising for nearly 2 years, fluctuating at 584–993 and 111–625 U/L respectively. Except for behavioral and language symptoms associated with ASD, the child appears normal in other aspects. The child's laboratory tests showed no abnormality, except that the serum levels of lactic acid was slightly higher than normal (1.89 mmol/L, normal 1.33–1.78 mmol/L). The child was prescribed with a traditional Chinese medicine during the process and the serum creatine kinase MB isoenzyme level decreased dramatically to 111 U/L after the treatment. This study firstly recorded the serum creatine kinase levels and the MB isoenzyme in patients with autism spectrum disorder for nearly 2 years, indicating that patients with ASD may experience long-term increases in serum creatine kinase and creatine kinase MB isoenzyme, and that the traditional Chinese medicine decoction Xinfukang can temporarily reduce the serum creatine kinase MB isoenzyme level in patients. Nevertheless, the effect is not sustained. Therefore, it is of great importance to conduct long-term longitudinal studies so as to elucidate the potential mechanism responsible for long-term elevation of serum creatine kinase level.

## Background

Autism Spectrum Disorder (ASD) is a complex neurodevelopmental disorder characterized by defective communication capacity, social impairment, stereotyped behaviors, and limited interests (1). The latest prevalence of ASD in the United States is 2.3% (2). There are at least 78 million people with ASD which makes it one of the fastest growing diseases in the world (3). It was reported that in 2013, the prevalence of ASD was 0.7% in China (4). While ASD's pathogenesis is still unknown, recent studies have found that some ASDs may be associated with inborn errors of metabolism (5). Some metabolic disorders can only be identified by non-targeted biochemical markers (5) and serumcreatine kinase (CK) values may be one of the potential indicators to it (6).

The reversible transfer of high-energy phosphate bonds between creatine and ATP can be catalyzed by CK, also referred to as phosphocreatine kinase (CPK). It is a significant regulatory enzyme provides energy for muscle contraction (7). It exists in skeletal muscle, cardiac muscle, brain tissue and other tissues that require a large amount of energy supply (7). The creatine kinase MB isoenzyme (CK-MB) is one of the isoenzymes of CK, and the two are often combined as a laboratory indicator for clinical judgment of myocardial injury (8, 9).

However, the results of current studies regarding serum CK levels in patients with ASD are inconsistent. Some studies have found that serum CK levels in ASD patients are higher than those in healthy people. Hassan et al. (10) compared the serum CK values in 73 ASD men and those in 73 age-matched healthy men, and revealed that the serum CK value in the ASD group was greater than that of the healthy control group (*P* < 0.05) (the serum CK value was 137 ± 12.96 U/L). It is consistent with the findings of El-Ansary et al. (11). Nonetheless, this study did not take into account the gender difference of patients and the highest serum CK value in the ASD group was only 63 U/L. Other studies found that not all ASD patients exhibit elevated serum CK levels. According to Mosalem et al. (12), the serum CK value of ASD children was 72.35% higher than that of normal children, with a serum CK value of 284.277 ± 242.477 U/L. A retrospective study conducted by Polling et al. (13) found that only 47% of ASD patients had abnormally elevated serum CK values. Cohen et al. (14) found that there are no differences in serum CK levels between children with ASD and normal children (*P* > 0.05). However, none of these studies discussed the serum CK-MB.

For traditional Chinese medicine therapy of patients with ASD, the choice of medicine is mainly based on the experience of physicians for which there is no unified standards to refer. Choosing traditional Chinese medicine based on tongue manifestations, pulse manifestation and clinical symptoms can improve symptoms associated with ASD (15). In particular, it can significantly improve patients' verbal communication ability and social communication ability, whereas its effect on improving patients' stereotyped behaviors is poor (16). Traditional Chinese medicine decoction Xinfukang is used for heart failure in clinical practice and other cardiac diseases. Although there were no studies focusing on the use of traditional Chinese medicine decoction Xinfukang for ASD patients, a study including 150 children with elevated serum level of CK-MB showed that traditional Chinese medicine Xinfukang can better reduce the serum levels of CK-MB of pediatric patients, compared with the western medicine group (oral vitamin C, vitamin E, inosine tablets and coenzyme Q10 for intramuscular injection) (17). In an animal study, traditional Chinese medicine Xinfukang administered orally by gavage can decrease the serum levels of CK-MB in rats with exercise-induced myocardial injury (18).

Though the serum CK levels were reported to be elevated in ASD patients, no report mentioned that the serum CK levels exceeded 900 U/L. There is a paucity of previous study focusing on the serum CK-MB levels in ASD patients, and there has been no report on the simultaneous monitoring of serum CK and CK-MB for 2 years continuously, making this research unique in this field. The case of an ASD child with abnormally elevated serum CK and CK-MB levels for almost 2 years, fluctuating between 584 and 993 (reference range, normal 29.0–168.0 U/L) and 111–625 U/L (reference range, normal 0.0–24.0 U/L), respectively, is described in this article. Apart from ASD-related behavior and language performance, the child is symptom free. During this period, the child was treated with the Chinese herbal decoction Xinfukang. After the treatment, the serum CK-MB level decreased to 111 U/L, but then increased again.

## Case presentation

**Figure 1:**
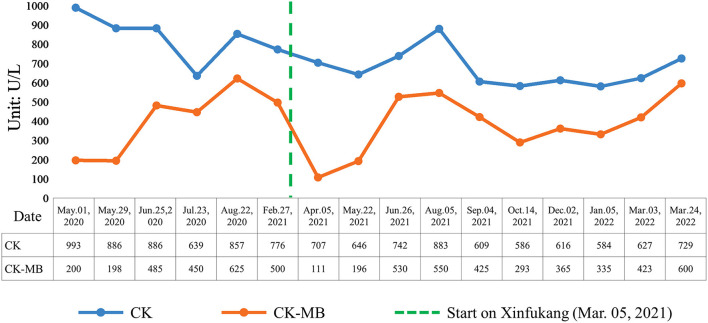
Changes of serum CK and CK-MB levels in the child.

A 5-year-old female Asian child with communication disorder, stereotyped behavior, and limited interest was diagnosed with ASD. The patient had no history of epilepsy or febrile seizures. The parents of the child are healthy, without any history of genetic disease in the family. The mother was healthy during her pregnancy, and the child is a first-born only child. The vaccination was administered on time and no adverse reactions were reported. She is a child with normal limb development, good nutrition, normal gross motor development, and delayed fine motor development. By the age of 2, parents observed that the child refused to communicate with others, showing a lack of eye contact, no responded to names, poor language skills, confusion or repetition of words, self-talk, rigid language, occasional strange noises, no response to teaching, not responding to commands, limited interest, pacing back and forth, irritability, and night terrors. The child's Autism Behavior Rating Scale (ABC) score was 132, while the Childhood Autism Rating Scale (CARS) score was 35. The child's doctor diagnosed her with ASD. The prescribed medications for her were Lactulose 10 ml/day, Singulair 4 mg/day, Cetirizine 2.5 mg/day, and Folic acid 5 mg three times per day. The child's serum CK value was 993 U/L and the CK-MB value was 200 U/L when he was 3 years old (May 2020), both of which were higher than normal. Besides, the results of electrocardiogram, liver and renal function tests and cardiac color ultrasound were normal. Upon multiple rechecks, serum CK and CK-MB values were higher than normal (see Figure 1). The results of electrocardiogram, cardiac color ultrasound and liver and renal function tests were normal. Serum level of folic acid showed unremarkable, serum level of lactate acid was 1.89 mmol/l, slightly higher than normal (normal 1.33–1.78 mmol/l). We performed whole exome genetic screening, blood genetic metabolic disease amino acid and acylcarnitine, urine organic acid comprehensive analysis, troponin, myoglobin, rheumatoid factor, antinuclear antibody, C-reactive protein, antistreptolysin “o” test, myositis-specific antibodies tests (including anti-KU antibody, anti-JO-1 antibody, anti-RO-52 antibody, anti-PM-SCL75 antibody, and anti-MDA5 antibody) and other related tests. As a result of all the tests, there are no abnormalities. Nevertheless, muscle biopsy, a serum lymphocyte test, and a platelet mitochondrial test were not performed due to parental refusal. The child showed no symptoms of fever, palpitation, chest pain, suffocation, cyanosis, muscle pain, or changes in muscle tone throughout the entire process. Based on the above examinations and clinical manifestations, doctors ruled out possible causes such as myocardial damage, liver damage, myositis, juvenile idiopathic arthritis, hypokalemia, and hypothyroidism.

**Figure 2:**
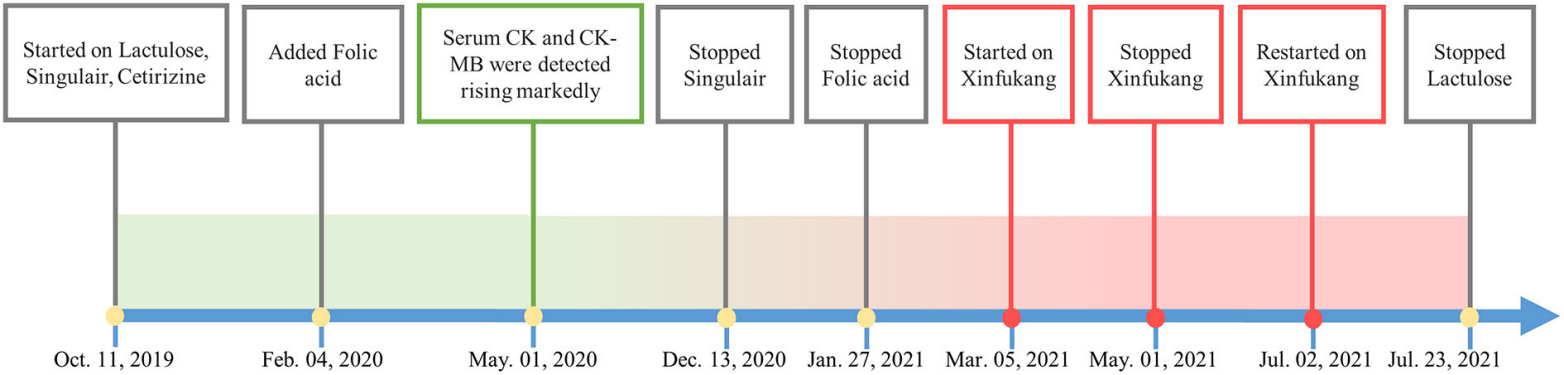
Medication history.

During the periods of treatment, western medicine seemed to has no effect on serum CK and CK-MB level of the child with ASD. Thus when the child aged 4 (March 2021), her parents asked physician to add traditional Chinese medicine to the treatment regimens and physician gradually decreased the use of other medicine and added traditional Chinese medicine decoction Xinfukang according to clinical manifestations and tongue manifestations, pulse manifestations (see Figure 2). Traditional Chinese medicine was decocted with water for 30 mins, and the decoction was administered about 150 ml each time orally. Within 1 month of oral treatment, the serum CK-MB level of the child decreased to 111 U/L, and the serum CK value decreased to 707 U/L (see Figure 1). In May 2021, the parents discontinued taking traditional Chinese medicine on their own since the child caught a cold. This child took the traditional Chinese medicine decoction Xinfukang for treatment again in July 2021, and the decoction was taken orally until the end of the monitoring. No side effects were observed during the period. Unfortunately, the serum CK and CK-MB levels of the child were not present significantly decreased.

During 2 years of monitoring and treatment, the doctor observed that the child's language comprehension and expression abilities occasionally regressed, while the overall trend was improving. The number of night terrors gradually decreased and eventually disappeared. At the end of the study, the child's ABC volume represented “60 points” and the CARS volume represented “34 points.” The parents of the child believe that the child is displaying enhanced social skills, with more eye contacts with others. Moreover, the child is able to express simple ideas through language or gestures, and can complete some simple command actions. It has been shown that Chinese medicine has a significant effect on improving moods and sleep in the child. The child's teacher believes that the child's attention has improved. However, neither the physician nor the child's parents thought that the child's behavior, language development, mood, sleep, and other aspects were not related to serum CK and CK-MB. For instance, the child's parents noted that when the child visited the doctor on August 22, 2020, the child showed improved comprehension and more stable mood in that month, but with relatively high serum CK and CK-MB levels were relatively high, at 993 and 625 U/L, respectively. During January 2022, the child was irritable without obvious inducements, and his comprehension abilities were poorer than before, though the serum CK levels did not fluctuate significantly in comparison to before (see Figure 1). Therefore, it appears that serum CK and CK-MB levels are not associated with the condition of children with ASD.

## Discussion

The serum CK and serum CK-MB are often used in combination as laboratory indicators of myocardial injury (8, 9). In addition, the elevation of serum CK levels can also be seen in hereditary or acquired myopathies such as muscular dystrophy, metabolic myopathy, congenital myopathy, drug- or toxic- induced, inflammatory myopathy, and endocrine myopathy, etc. (19). Serum CK elevation can also be caused by non-myopathy factors, such as getting black, high fever, strenuous exercise, trauma, viral infection, malignant hyperthermia, and idiopathic hyperckemia (19, 20). Neurological diseases such as epilepsy (21), schizophrenia (22), etc. may increase serum CK levels. According to previous studies on adult psychosis, acute psychosis can cause elevated serum CK levels, whereas chronic psychosis patients present normal serum CK levels (22).

CK-MB is a hybrid dimer composed of M-type and B-type monomer subunits, which has reversible catalytic functions for creatinine and ATP phosphorylation (23). In normal serum, CK-MB constitutes less than 5% of CK (24). It is generally believed that serum CK-MB/CK ≥ 6% indicates myocardial injury, and that CK-MB activity can also be increased in skeletal muscle disease. However, serum CK/CK-MB is less than 5% in most cases (25). The possibility of myocardial injury in the child is ruled out based on her clinical symptoms and the outcomes of related laboratory tests despite the serum CK-MB/CK of the child described in this article being higher than 6%. Xiao et al. (24) found that patients with traumatic brain injury or brain diseases may have significantly elevated CK-MB and that in some cases, even the inversion of CK-MB and CK measurements would emerge. Although the serum CK-MB level of this child did not exceed the serum CK level, its significant increase may be related to autism.

It is unclear what causes the rise in serum CK levels in ASD. However, it has previously been explained in terms of mitochondrial dysfunction. In ASD patients, mitochondrial dysfunction is a common metabolic disorder (5). In their study, Rossignol et al. (26) argue that mitochondrial dysfunction can result in the loss of cellular integrity in specific organs, including muscle and liver, enabling the release of CK into the bloodstream, leading to increased serum levels of CK. However, not all ASD patients with mitochondrial dysfunction have elevated serum CK levels. In their study, Frye et al. (27–29) found that only a minority of ASD patients with mitochondrial disease had elevated serum CK levels. In a study of 25 patients who had ASD and mitochondrial dysfunction, it was found that only 32% had elevated serum CK levels (30). Furthermore, not all elevated serum CK levels are caused by mitochondrial dysfunction. According to a meta-analysis, 5% of ASD patients suffer from mitochondrial disease, while 47% of ASD patients have elevated serum CK, significantly higher than 5% (31). Therefore, further studies should be conducted to explore whether there are other potential mechanisms responsible for the elevation of serum CK level in patients with ASD.

Muscle biopsy is frequently used to detect mitochondrial diseases (32), as is mitochondrial examination of serum lymphocytes and plates can also be a laboratory indicator of mitochondrial disease (33). Lactate is a key biomarker for identifying mitochondrial disease (31, 34). The parents of the child described in this article refused muscle biopsy and serum lymphocyte and platelet-related mitochondrial tests. The child's serum lactate acid level was 1.89 mmol/L, slightly higher than normal. Studies have shown that children with ASD and mitochondrial disease may suffer from fatigue, gastrointestinal problems, abnormal types of neurodevelopmental degeneration, seizures, and motor retardation (35). However, these symptoms were not observed in the patient described in this article. Further, over a period of nearly 2 years, the doctor monitored the child's serum CK level has been rising, which is inconsistent with the intermittent increase in serum CK levels in ASD patients with mitochondrial disease previously reported by Weissman et al. (30). Therefore, it cannot concluded that the increase in serum CK in this patient is linked to mitochondrial dysfunction.

Traditional Chinese medicine decoction Xinfukang can decrease the serum levels of CK-MB of rats with exercise-induced myocardial injury by decreasing the serum levels of cortisol and elevating the serum levels of testosterone which to protect myocardial cell (18). On the other hand, Xinfukang oral liquid has effects on mitochondrion; Qilin et al. (36) found that Xinfukang oral liquid can adjust mitochondrial proteomics in myocardial cells of rats with heart failure by upregulating the expression of α subunit of ATP synthetase related to energy metabolism which can improve energy metabolism disorder in myocardial cell to a certain degree and downregulate the expression of mitochondrial stress-70 protein in myocardial cell at the same time. Stress-70 protein family played an important role in preventing and treating cell damage caused by stress and repairing damaged cells. Xinfukang oral liquid can alleviate stress reaction and protect damaged myocardium by downregulating the expression of mitochondrial stress-70 protein. Physician expected to decrease serum levels of CK-MB of the child with ASD and adjust mitochondrial function to improve her clinical manifestations related to ASD by giving her Xinfukang. At the time of 1 month after the initial use of Xinfukang, serum levels of CK-MB of the child with ASD indeed decreased but increased again without subsequent improvement in spite of the continual use of Xinfukang. Luckily, however, language expression abilities and moods of the child with ASD seemed to be improved with the traditional Chinese medicine.

Patients with ASD may have elevated serum CK levels, which have previously been associated with mitochondrial dysfunction. The article first reported a child with ASD who had significantly elevated serum levels of CK and CK-MB for approximate 2 years and recorded the changes of serum CK, CK-MB in details. After the use of traditional Chinese medicine decoction Xinfukang, the decrease of serum CK-MB once appeared. However the effect was not persistent and the function mechanism remains unclear. As the case indicated, child with ASD may have the chronically elevated serum levels of CK and CK-MB and traditional Chinese medicine decoction Xinfukang can improve child's behavioral and language symptoms, moods and so on, but cannot improve the serum levels of CK and CK-MB in the long term. Due to no reports of similar cases in the past and no studies about traditional Chinese medicine decoction Xinfukang therapy for ASD, it is a huge challenge to diagnose and treat ASD. One of the limitation of this study is lacking of related evidence to support it. The next step should be the completion of a long-term longitudinal study of CK and CK-MB levels in ASD patients to clarify the potential mechanisms responsible for long-term elevation, so as to explore further the possibility that Xinfukang might be effective in treating ASD.

## Data availability statement

The original contributions presented in the study are included in the article/supplementary material, further inquiries can be directed to the corresponding authors.

## Ethics statement

Written informed consent was obtained from the individual(s), and minor(s)' legal guardian/next of kin, for the publication of any potentially identifiable images or data included in this article.

## Author contributions

PR and RM contributed to conception and design of the study. SZ wrote the first draft of the manuscript. QF, XZ, and MC wrote sections of the manuscript. LY and YS collected information about the patient's condition. All authors contributed to manuscript revision, read, and approved the submitted version.

## Funding

This work was supported by the National Natural Science Foundation of China (No. 30973772) and the Program for QiHuang Scholar (RM) in State Administration of Traditional Chinese Medicine.

## Conflict of interest

The authors declare that the research was conducted in the absence of any commercial or financial relationships that could be construed as a potential conflict of interest.

## Publisher's note

All claims expressed in this article are solely those of the authors and do not necessarily represent those of their affiliated organizations, or those of the publisher, the editors and the reviewers. Any product that may be evaluated in this article, or claim that may be made by its manufacturer, is not guaranteed or endorsed by the publisher.
